# Pathogenic postzygotic mosaicism in the tyrosine receptor kinase pathway: potential unidentified human disease hidden away in a few cells

**DOI:** 10.1111/febs.15528

**Published:** 2020-09-05

**Authors:** Irene Tiemann‐Boege, Theresa Mair, Atena Yasari, Michal Zurovec

**Affiliations:** ^1^ Institute of Biophysics Johannes Kepler University Linz Austria; ^2^ Biology Centre of the Czech Academy of Sciences Institute of Entomology Ceske Budejovice Czech Republic

**Keywords:** gain of function, pathogenic variants, penetrance, postzygotic mosaicisms, tyrosine kinase receptor

## Abstract

Mutations occurring during embryonic development affect only a subset of cells resulting in two or more distinct cell populations that are present at different levels, also known as postzygotic mosaicism (PZM). Although PZM is a common biological phenomenon, it is often overlooked as a source of disease due to the challenges associated with its detection and characterization, especially for very low‐frequency variants. Moreover, PZM can cause a different phenotype compared to constitutional mutations. Especially, lethal mutations in receptor tyrosine kinase (RTK) pathway genes, which exist only in a mosaic state, can have completely new clinical manifestations and can look very different from the associated monogenic disorder. However, some key questions are still not addressed, such as the level of mosaicism resulting in a pathogenic phenotype and how the clinical outcome changes with the development and age. Addressing these questions is not trivial as we require methods with the sensitivity to capture some of these variants hidden away in very few cells. Recent ultra‐accurate deep‐sequencing approaches can now identify these low‐level mosaics and will be central to understand systemic and local effects of mosaicism in the RTK pathway. The main focus of this review is to highlight the importance of low‐level mosaics and the need to include their detection in studies of genomic variation associated with disease.

## Graphical abstract

Gain‐of‐function mutations in the receptor tyrosine kinase pathway are often lethal; yet, it might be tolerated at very low frequencies as postzygotic mosaics resulting in a vast range of new phenotypes, unknown before the detection of the mosaic disorder. With the advent of ultra‐accurate next‐generation sequencing, these undercharacterized mosaics hidden in a few cells can now be better characterized in terms of their biological relevance in disease.

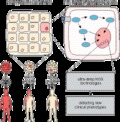

**Abbreviations**
DNM: denonbreakingspacenovo mutations
DS: duplex sequencing
FD/MAS: fibrous dysplasia/McCune–Albright syndrome
GPCR: G protein‐coupled receptors
NCM: neurocutaneous melanocytosis
NGS: next‐generation sequencing
OES: oculoectodermal syndrome
PPV: phakomatosis pigmentovascularis
PROS: PIK3CA‐related overgrowth spectrum syndromes
PZM: postzygotic mosaicism
RTK: receptor tyrosine kinase
SFMS: Schimmelpenning–Feuerstein–Mims syndrome
SWS: Sturge–Weber syndrome

## Introduction

*De novo mutations* (DNMs) occurring during postzygotic development end up only in a subset of somatic and/or germline cells resulting in a well‐described phenomenon known as postzygotic mosaicism (PZM) or also postzygotic variation. In mosaics, DNA changes were acquired from the zygote stage onwards throughout the lifespan. Thus, two or more distinct cell populations are present in mosaics at different levels in one or more tissues, depending on the time of occurrence of the DNM and the evolution of the mutation during development. Here, mosaicism is defined as the presence of genetically distinct lineages of cells in an organism derived from a single zygote (also reviewed in Ref. [1]). This is in contrast to DNMs occurring in the germline that are inherited constitutionally by all the cells in the offspring. If changes occur later in the development or in the adult, they might be confined to a single organ; for example, male germline mosaics are confined to the testis or cancer is a mosaic in somatic tissue [2].

Currently, we are saturated with next‐generation sequencing (NGS) data exploring the relationship between genotype data and disease using genome‐wide association studies (GWAS) or exome sequencing; yet, in many cases the observed phenotype cannot be pinpointed to specific constitutional variants. This ‘missing heritability’ shaping differences between individuals is often attributed to other factors, including gene regulation and environment, or genetic constitutional variants with weak effects missed by GWAS with insufficient power. However, it is also possible that a phenotype or disease is caused by genetic variation within a few cells carrying the alternative allele that are missed by standard sequencing approaches [3, 4, 5, 6]. This is the case for variants only viable in a mosaic state with a small fraction of the screened cells with the alternative allele being causal for the observed phenotype.

Within the last decade, PZM has been recognized as an important factor explaining disease. However, there are several limitations in our knowledge about PZM disorders: the nature and relationship between the mutation and the clinical outcome (genotype/phenotype), which molecular diagnostic tools should be used, which tissues should be assayed, and the best methods to capture low‐level mosaicism and their limitations. Mosaicism can potentially cause a different phenotype compared to constitutional mutations affecting the majority of the cells. This is complicated by the fact that patients with PZMs caused by the same mosaic mutation might not look alike, a unique challenge for clinicians, who seek a unified approach to identify the disease [2, 7].

In this review, we will address some of these aspects with focus on mosaicism of activating mutations in the receptor tyrosine kinase (RTK) pathway. Some gain‐of‐function mutations in this pathway are only viable in the mosaic state rendering completely new phenotypes and diseases. The main focus of this review is to highlight the current state of the art on these mosaics and the new opportunities to study these archetypical PZMs. In addition, our goal is also to convince the readership that the design of genomic variation studies should include the detection of low‐level variation that could be key in explaining the observed phenotype. Other reviews have focused on more general aspects of PZM including hematopoietic mosaicism and loss of chromosome Y (e.g., see Ref. [1]) and will not be addressed here.

## PZM and selfish mutations

Mutations can be triggered by distinct environmental factors, but also by replication errors, or spontaneous DNA lesions. Recently, one of the major mutational mechanisms driving genetic mosaicism in humans has been described as oxidative stress and spontaneous deamination of methylated cytosines [8]. How these mutant lineages expand or disappear in healthy tissues during the development has been a highly active research area in the last years [6, 9, 10, 11, 12, 13, 14, 15] and fits within the neutral theory of mutagenesis and genetic drift. However, in this review, we will focus on a unique type of mutagenesis: point mutations in the RTK and its pathway components (e.g., *RAS*) that change the function of the protein and lead to the clonal growth of the cell.

Mutations have the potential to lead to a broad range of cellular phenotypes and can affect the relative fitness of a cell. Most mutations are either neutral or decrease the fitness relative to wild‐type cells, if occurring in a functional region of the genome. In contrast, some mutations can lead to a proliferative advantage of the cell resulting in the clonal expansion in affected lineages. Examples of such advantageous or ‘selfish’ mutations in the male germline have been documented in some RTK genes (e.g., *FGFR3* and *FGFR2* [16, 17, 18]) and components of their downstream signaling pathway such as *PTPN11*, *HRAS,* and *KRAS* [19, 20]. Cell growth may occur by a larger number of cell divisions (increasing the cell mass) or by suppression of apoptosis. Moreover, a certain mutation that causes clonal expansion might interfere with cell differentiation, so that the same mutation might have a different outcome throughout the development and in different somatic tissues, as, for example, described for *PIK3CA*‐associated mosaics [21].

Mutations in a handful of genes such as *FGFR2*, *FGFR3*, *HRAS*, *PTPN11*, *KRAS,* and *RET* [17, 18, 22, 23], and recently described genes such as *BRAF*, *CBL*, *MAPK1*, *MAPK2*, and *RAF1* [20], all part of the RTK signaling pathway (*RTK/MAPK/RAS*) have been demonstrated to expand in the aging testis and represent tissue‐restricted mosaics [16, 17, 18, 20, 22, 24, 25]. These mutations are mainly missense mutations that modify the signal modulation of the RTK pathway (usually by a ligand‐independent activation of the mutant protein) that affects cell survival and/or cell fate. It has been observed that cells in the male reproductive system carrying activating mutations grow into mutant microclusters of spermatogonial stem cells that become larger with age [17, 18, 20, 22, 26, 27, 28]. As a result, the germline becomes a mosaic for several different RTK mutations as men age, all in different anatomical locations of the testes, as shown for different mutations, suggesting that these mutations arise and expand independently [19, 29].

Activating RTK mutations can also expand during zygotic development and have been described in the context of PZM. Yet, given the importance of the RTK pathway in the development, it is difficult to predict the clinical manifestation of a mosaic mutation since the increased signal activation might result in different pleiotropic effects in terms of cell growth, differentiation, and apoptosis. Moreover, very strong activating mutations might have disruptive effects and be tolerated only in certain tissues or at different amounts in a few cells. For example, strongly activating mutations in *HRAS* that are highly prevalent in cancer, hardly overlap with germline mosaics [24, 25, 30]. Thus, it is not surprising that the same PZM mutant might result in highly different phenotypes. To date, it is still unknown how common activating RTK mutations are during postzygotic development, which is unfortunate because this could be an important mechanism linked to uncatalogued diseases.

## PZM diseases linked to the receptor tyrosine kinase pathway

**Fig. 1:**
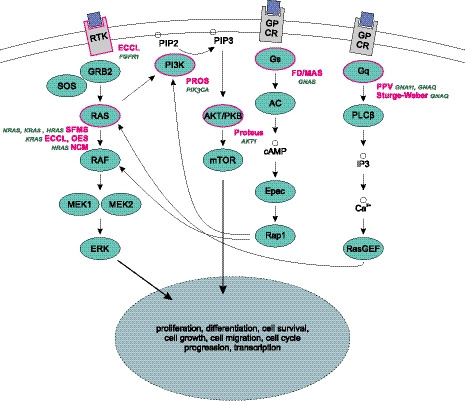
PZM diseases linked to the RTK pathway. Shown are the RTK and G protein‐coupled receptors (GPCR) and the actors of their downstream signaling cascade involving the RAS/MAPK/Erk pathways, as well as the interaction between these pathways. Activation of GPCR and RTK leads to a cascade of intracellular signals involving many different genes that finally regulate important cellular processes such as cell growth, differentiation, migration, and many more. Genes affected by postzygotic mosaic mutations causing disease (magenta border), the associated syndromes (magenta), and the specific genes affected (green) are also shown.

Mosaicism is increasingly recognized as a cause of developmental disorders with a wide spectrum of phenotypes and clinical outcomes. Especially, somatic mutations in genes of the RTK pathway and downstream signaling *RAS/MAPK/Erk* (e.g., *PI3K/PTEN/AKT/TSC/mTORC1*) expressed in specific organs may result in a spectrum of different phenotypes ranging from isolated small lesions with minimal or no overgrowth to extensive lesions and tumor susceptibility (Fig. 1). In fact, mosaicism for monogenic disorders was postulated as an explanation for the patchy manifestations of Mendelian disorders and lack of familial recurrence of activating mutations in genes of the *RTK/MAPK* signaling pathway. Here, we discuss selected entities caused by PZM [Proteus syndrome, *PIK3CA*‐related overgrowth spectrum syndromes (PROS), fibrous dysplasia/McCune–Albright syndrome (FD/MAS), Sturge–Weber syndrome (SWS), and mosaic RASopathies with cutaneous manifestations] with focus on the patterns of disease (gene, level of mosaicism with clinical manifestations, affected tissue, and time of onset if known). With very few exceptions, all PZM diseases are only viable in the mosaic state (see also Table 1 and Fig. 1).

**Table 1:** Syndromes caused by mutations in RTK genes documented as pathogenic mosaics.

Syndrome	Genes and substitutions	Level of mosaicism	Affected tissue	Time of onset	Ref.
Proteus	*AKT1* (E17K)	Level and phenotype of Proteus greatly depend on the timing of the mutation, the affected tissue type, or restriction to certain germ layer.	Asymmetrically and irregularly growing tissues mainly in skin, bone, and adipose tissue; increased risk in thrombosis and subsequent pulmonary embolism	6–18 months after birth	[31, 64]
PROS; for example, Klippel–Trenaunay	*PIK3CA* (E545K, E454G, H1047R, H1047L)	< 1% detected by NGS	CLVM (capillary, lymphatic, and venous malformations), skin and tissue with lesions, and buccal cells	Phenotype dependent on mutation type and distribution	[32, 65]
FD/MAS	*GNAS* (R201H, R201C)	Unknown; ‘obligatory mosaic’	Endoderm, mesoderm, and ectoderm	Children and young adults	[66]
SWS	*GNAQ* (R183Q)	Mutant allele in affected tissues ranged from 1% to 18%	Skin specially face, eye, nervous, and neurological anomalies	Infants	[35]
PPV and extensive dermal melanocytosis	*GNA11* (R183C, R183S) *GNAQ* (R183Q, Q209P)	Low level of postzygotic mutations; percentage of mosaicism in skin was lowest with 1.5%	Dermal melanocytosis (Mongolian blue spots), ocular melanocytosis, vascular birthmarks, and neurological abnormalities	Infants	[41, 67, 68, 69, 70]
NCM	*NRAS* (Q61R, Q61K)	Affected cutaneous and neurological tissues	Development in neural crest and neuroectoderm; skin and central nerve system	Children	[36, 37]
SFMS	*HRAS* (G13R) *KRAS* (G12D) *NRAS* (Q61R)	Mutation frequency of 52% in head nevus sebaceous, 13% in hyperpigmented lesions, and 24.3% in lip nevus sebaceous tissues	Nevus sebaceous, neurological anomalies, eye, skeletal, height, brain, head, genitourinary, cardiovascular, neoplasia	From birth onwards	[38, 39]
OES	*KRAS* (A146T, A146V, G13D, L19F)	RASopathy; frequency < 40% of tissues	Skin; epibulbar dermoids and congenital scalp lesions (aplasia cutis congenita; ACC)	Children	[71, 72]
Encephalocraniocutaneous Lipomatosis (ECCL)	*FGFR1* (K656E, N546K) *KRAS* (A146T)	Alternate allele fraction of 23–55% in fibroblasts from affected tissues; not detected in saliva or blood	Cutaneous, ocular, and CNS; nevus psiloliparus as hallmark of ECCL	Children	[71, 73, 74]

We start with the Proteus syndrome, one of the archetypal mosaic disorders, and the PROS syndrome that has a very patchy distribution of features reviewed in Ref. [31]. Proteus is an extremely rare disease (< 1 in 10 million) with mosaic mutations in *AKT1* that, if constitutional, are lethal. The disease is characterized by asymmetrically and irregularly growing tissues anywhere in the body, but is observed mainly in adipose tissue, skin, and bone. Patients are usually born without having a significant phenotype, but then start with asymmetric overgrowth at the age of 6–18 months. The specific phenotype depends on the timing of the mutation, the affected tissue type, or whether the mutated cell was restricted to a certain germ layer [31].

Postzygotic mosaicism with similar characteristics as Proteus are some *PIK3CA* gain‐of‐function mutations, classified as strongly, intermediately, or weakly activating, resulting in a range of pediatric developmental phenotypes described under the umbrella term of PROS syndromes. These disorders are characterized by cutaneous vascular malformations with segmental overgrowth and involve multiple tissues or body regions, producing, for example, congenital lipomatosis with overgrowth, vascular malformations, epidermal nevi, and skeletal abnormalities. Klippel–Trenaunay syndrome is one of the PROS syndromes caused by somatic gain‐of‐function mutations in *PIK3CA* that activates the *PI3K/AKT/mTOR* pathway and results in dysregulation of cellular growth. The clinical outcome includes cutaneous port‐wine stains, tissue hypertrophy, and varicosities, as well as overgrowth of capillary, lymphatic, and venous malformations in lower and also upper limbs in children or young adults with the phenotypes changing over time [32].

Another quite interesting mosaic disease is the FD/MAS because it is an ‘obligate mosaic’, which means that a mutant cell survives only in the context of wild‐type cells. In FD/MAS, missense mutations in *GNAS* (R201H or R201C) lead to the mosaic activation of Gα5 and thus impaired intrinsic GTPase activity leading to ligand‐independent signaling and production of excess intracellular cAMP [33]. The incidence of mutations varies but can involve tissues from all three germ layers (endoderm, mesoderm, and ectoderm). The phenotype of FD/MAS involves any part of the skeleton and may be associated with highly variable cutaneous, endocrine, and other extraskeletal features. Remarkably, some mutations result in a disorder only in the context of a mosaic, where some cells carry the dysfunctional mutant, as was shown in xenographs implanted in mice, in which the typical FD lesions developed only if mutant cells were in the presence of wild‐type cells [34]. The importance of wild‐type cells for lesion progression remains an unknown question and could be addressed with animal models like *Drosophila*, as described in a different section. Moreover, the effect of mosaicism varies in different tissues and may be related to the tissue‐specific sensitivity of cAMP dysregulation or the intolerance of specific cell types to an overactivation of the Gα5 receptor. The SWS is also caused by a mosaic gain‐of‐function mutation in *GNAQ* that activates the signal‐regulated kinase (ERK), which in turn signals to MAPK increasing cell proliferation and/or inhibiting apoptosis [35]. The level of mosaicism in the affected tissues ranged from 1% to 18% in the majority of SWS patients. The mutations mainly affect the skin, although vascular malformations or venous‐capillary abnormalities are also observed [35].

A series of PZM affects mainly the skin, but can also be accompanied by a series of pleiotropic effects in the nervous system. Most of these PZM diseases are caused by mutations in *RAS* and are linked to ‘RASopathies’. Examples of this type of mosaicism are neurocutaneous melanocytosis (NCM), Schimmelpenning–Feuerstein–Mims syndrome (SFMS), phakomatosis pigmentovascularis (PPV), extensive dermal melanocytosis, and oculoectodermal syndrome (OES). NCM is a rare disorder characterized by mutations that constitutionally activate *NRAS* (Q61R, Q61K). Its clinical outcome is characterized by large or multiple melanocytic nevi on the skin, meningeal melanocytosis, or melanoma. Mutations causing NCM develop from the neural crest and neuroectoderm and are found in the affected skin, but not in the blood [36, 37]. SFMS is caused by autosomal dominant mutations in *NRAS* (Q61R), *HRAS* (G13R), and *KRAS* (G12D) that get manifested only as somatic mosaicism and are otherwise lethal [38, 39, 40]. The phenotypical outcome of the mutations ranges from epidermal nevus syndrome to neurological manifestations visible before 1 year of age that can also be accompanied by eye abnormalities or epilepsy. Benign or malignant tumors can also develop at later stages. The lesions are present only in skin tissue (nevus sebaceous) [39, 40], although they could extend to extracutaneous tissues [41]. For further information on other PZM‐causing diseases in *NRAS* or *KRAS*, see Table 1.

## Factors affecting the clinical outcome of PZM

### Are increasing levels of mosaicism proportional to the pathogenicity of the phenotype?

**Fig. 2:**
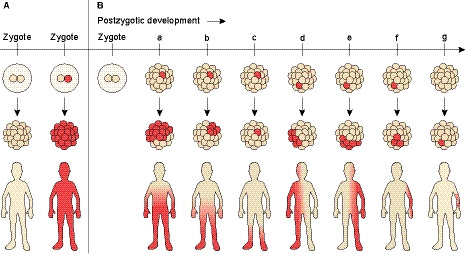
Development and manifestation of postzygotic mutations. (A) Left side shows a ‘normal’ zygote compared to a zygote with a dominant germline mutation (red) at the right side that is either transmitted from one of the parents or arose in one of the parental germ cells *de novo* and affects the whole body of the offspring. (B) In contrast, mutations can also arise postzygotically during embryonic development or throughout the life affecting only subsets of cells (PZM). In theory, an early mutation should lead to disseminated mosaicism involving more tissues of the body depending on the time of onset and specific layer (a‐e), whereas a later, lineage‐specific error should result in organ‐confined mosaicism subsequent to clonal expansion (e.g., skin lesions shown for case g).

Generally speaking, PZM has a milder clinical manifestation than inherited constitutional mutations present in all somatic cells (Fig. 2). This is well illustrated for strongly activating constitutional or germline mutations, for example, in *AKT1* and *PIK3CA* which are lethal; however, the same mutations in the context of PZM are viable and get manifested by different overgrowth phenotypes ranging in severity from slightly enlarged digits to gigantic limbs, or benign focal overgrowths [42]. The resulting phenotype of the mosaic and its clinical outcome depends on the number and organization of abnormal cells in relation to normal cells and how the mutation affects the cellular function, as, for example, it was described for cutaneous mosaicism [43]. Furthermore, the correlation between a potentially pathogenic phenotype and increasing levels of mosaicism is more likely in monogenic disorders caused by gain‐of‐function mutations like the activating RTK mutations (Fig. 2). The clinical manifestation also depends on whether the PZM occurs before or after cell differentiation events. For example, a *PIK3CA* mutation that occurs prior to germ layer differentiation might be manifested as a multisystem disease in PROS with cortical abnormalities, if derived from the ectoderm, or as capillary malformations, if derived from the mesoderm [44]. In contrast, a PZM localized to a specific region of a somatic tissue will produce disease manifestations restricted to tissue type and/or a segment of the body, as shown for Proteus syndrome [45].

Here, it is important to consider the consequences of the signal activation of the RTK pathway and the associated lethality of a mutation. The resulting phenotype depends on the activating strength of the mutant protein, where highly activating RTK mutations are usually lethal, as described for constitutional mutations in FGFR3 reflecting well this activation/severity of phenotype correlation: Disorders such as hypochondroplasia or achondroplasia are caused by mildly activating mutations, whereas the mutation causing thanatophoric dysplasia II is strongly activating and thus embryonically lethal (revised in Ref. [29, 30, 46]).

Every mutation causing a monogenic disorder can also occur in mosaic form, but in a mosaic context, the correlation between phenotype/activation is not as straightforward. While mosaics of mild or nonlethal mutations typically manifest themselves as a milder or atypical form of the monogenic disorder, strong activating or lethal mutations might produce unique phenotypes. The increase in phenotype severity for mildly activating mutations is exemplified in *PIK3C*A mutations that correlated fairly well with clinical and molecular features and the activation strength of the mutant protein. As such, a clonal‐focal overgrowth and predominant brain overgrowth were observed for highly activating mutations, but for less activating mutations, less severe somatic overgrowths and intermediate phenotypes were observed [21, 31].

In contrast, mosaicism for lethal mutations is usually tolerated only at very low frequencies and this might vary with the tissue and developmental stage. Very strong activation mutations might require only a smaller number of affected cells to show a clinical manifestation. Moreover, highly activating mutations might also be quickly eliminated during differentiation or might survive only as ‘obligate mosaics’, as exemplified for some gain‐of‐function mutations in *GNAS* (associated with FD/MAS) [33]. Thus, mosaicism of lethal mutations can render completely different phenotypes and cannot be deduced from a known disorder caused by the constitutional variant. For example, mutations in *RAS* genes (*HRAS*, *KRAS,* or *NRAS*) are often lethal; yet, the same mutation leading to mosaic RASopathies often leads to completely new phenotypes, unknown until the characterization of the mosaic variant. Such is the case for mutation of Q61R in *NRAS* that leads to either Schimmelpenning–Feuerstein–Mims (SFMS) or NCM as shown in Table 1.

### Age dependency

The long‐term clinical outcome of a mosaic might also change with age, with the size of the mosaicism increasing with age. Why does mosaicism increase with age? Under neutrality, frequencies of mutant variants increase with age as a result of a reduction in DNA repair activity, an increase in the incidence of replication errors, and/or random drift [14]. Note also that the somatic mutation rate is 4–25 times higher than the estimated germline rate [47]. Under a selection model, the proliferative advantage of the cells conferred by the mutation induces clonal growth such that clones become larger with time to detectable levels [48]. Thus, more clones are expected to accumulate with age and increase in size.

Multiple recent large‐scale studies have revealed that healthy individuals can also harbor mosaic mutations; the frequencies are low in young individuals, but can increase to detectable frequencies between 0.1% and 20% for individuals at mid‐age (30–50 years) or older [6, 9, 10, 11, 12, 13, 14, 15]. A study of hematopoietic stem and progenitor cells estimated an accumulation of PZM of ~ 0.13 single‐nucleotide changes per exome per year [49], also reviewed in Ref. [1]. Also for PZM diseases, mutations are acquired early in embryogenesis with lesions growing over the first years of life until they become apparent during childhood and adolescence, as is the case for FD/MAS [33].

In recent years, reports of rare mosaics or very low PZM detected with ultrasensitive methods have provided a more comprehensive picture of PZM. A recent study of the *TP53* gene that examined different tissues from babies to centenarians identified ultralow‐frequency mosaic clones already at a very young age with specific signatures identified also in older individuals indicating a lifelong expansion [13, 50]. However, it was also noted that the peripheral blood of a centenarian showed an unexpected low diversity of mutations, suggesting that some lineages disappear with age [13]. The more thorough characterization of the landscape of somatic mutations in protein‐coding genes in unaffected tissues will provide important insights into the mechanisms of age‐associated mutagenic processes, also associated with neutrality or selection.

## Technical challenges studying mosaicism

A major challenge for the study of PZM is distinguishing biologically relevant, low‐frequency postzygotic variants from technically induced errors. In previous years, only 1% of subjects younger than 50 years of age showed evidence for somatic point mutations [51, 52, 53]. Large clones resulting in frequencies of ~ 10% are detectable by standard whole‐exome or multigene NGS in, for example, clonal hematopoiesis [52, 53]. Alternatively, PZM is detected if the clones constitute a sizeable percentage of cells in confined biopsies [54]. However, standard depth (10×–30×) NGS methods of whole‐exome or targeted sequencing are not accurate enough to detect low‐level mosaics (below a frequency of < 5–10%). Increasing the sequencing depth to 10 000×–100 000× does not solve the problem either, since standard NGS has a background error rate of up to 1% precluding confident measurements of minor allele frequencies below 1%. These appear as ‘background noise’ in most genetic assays, and standard bioinformatic filters frequently miss them.

Over the past years, studies with the required sensitivity to measure PZM in normal or unaffected somatic tissue are picking up with the development of ultrasensitive sequencing technologies. These involve special library preparation protocols to distinguish a real mosaic from artifacts (e.g., Ref. [9, 13]). These higher accuracy NGS technologies, for example, molecular inversion probes or amplicon sequencing, can detect mutations at lower frequencies (< 1%) and resolve lower‐frequency mosaicism or subclones [9, 15]. Currently, the most accurate NGS method for detecting ultralow variants is duplex sequencing (DS), which uses barcodes to retrieve the sequence of both strands of the DNA sequence [55, 56] and reviewed in Ref. [57]. Using ultra‐accurate DS, it was shown that low‐frequency (0.1–0.01%) *TP53* mutations exist in multiple healthy tissues, from newborn to centenarian [13, 58]. More importantly, using this highly sensitive method also allowed to identify low‐frequency *TP53* mutations that were heavily enriched in women with ovarian cancer, but not in unaffected women, highlighting the importance of this sequencing method to identify mosaic mutations correlated with disease [13].

The precise choice of which tissues or cells to select for collection is also an important concern. These should represent tissues from different developmental lineages (endoderm, mesoderm, and ectoderm) and should be easy to retrieve. Blood, fibroblasts, saliva, and urine are easy to sample and contain the major components of mesodermal origin, whereas buccal swaps or skin biopsies represent the ectoderm. When screening for PZM with a clinical outcome, the best approach is to sample small biopsies of the affected tissue, as well as biopsies of normal tissue surrounding the lesion [21].

## *Drosophila* may serve as a model of PZM in humans

*Drosophila* has proven to be an excellent ‘mosaic model’ for research into cancer mechanisms, regenerative growth, stem cell behavior, and cell competition, as it allows the study of PZM in more detail, such as the microenvironment of overgrown tissues or modifiers affecting such overgrowth. Moreover, the experimental induction of mosaics in *Drosophila* has been used for many years as a research tool to characterize lethal genes, to monitor the growth of tumor cell clones or to investigate cell competition mechanisms. In particular, mosaicism in the *RAS* signaling pathway has been studied very intensively in *Drosophila* mosaic clones of imaginal disk cells carrying the activating *RAS^Val12^* mutation that forms benign tumors or slight overgrowth (see Ref. [59], for a recent review).

Another advantage of this system is that mosaic clones can be induced tissue‐specifically, both in the soma and in the germline, and allows to examine the fate of the individual PZM in different tissues or clone sizes. This information can then be translated into the penetrance of the PZM, the cell autonomy, and the pleiotropic effect of the mutation in developmental time or aging. Similarly, it is possible to prepare flies with a certain constant proportion of mutant clones on different genetic backgrounds and search for genetic modifiers of disease severity. In addition, *Drosophila* can be used to find ‘non‐standard phenotypes of some mosaic clones’. For example, several recessive mutations in the *Drosophila BMPR1A* gene in heterozygous clones induced in wild‐type individuals caused wing damage. This phenotype was not observed in individuals consisting of heterozygous cells or in heterozygous individuals carrying homozygous mutant clones. Similar heteroclones with a phenotype resulting from a ‘one‐hit’ mutation could also occur in humans [60].

Finally, *Drosophila* PZM models may be useful for studying physiological phenomena difficult to perform in human tissues or mammalian models—for example, organ growth associated with cell competition is known to eliminate clones containing cells with lower fitness [61]. Such experiments may bring relevant implications for humans; however, genetic differences between *Drosophila* and humans may pose a challenge for the translation of such data to biomedical research [62, 63].

## Conclusions

It is widely acknowledged that very low‐frequency mosaic mutations have been undercharacterized. This is added to the difficulty that the genetic profile of a single tissue collected at one time point is not a faithful portrait of other tissues from the same subject or the same tissue throughout the lifetime of the individual. Yet, ultra‐accurate NGS technologies have opened the door to unravel the precise molecular role of low‐frequency PZM in somatic diseases and could tackle one of the big remaining questions: Which PZMs accumulate randomly with age and which are causal of disease?

## Conflict of interest

The authors declare no conflict of interest.

## Author contributions

ITB organized, structured the contents, edited the manuscript, and wrote parts of the manuscript. TM and AY researched the PZM diseases and wrote parts of the manuscript. TM designed the figures and the tables. MZ wrote the section about Drosophila.
